# Synovial Chondromatosis of the Subacromial Bursa Causing a Bursal-Sided Rotator Cuff Tear

**DOI:** 10.1155/2015/259483

**Published:** 2015-03-12

**Authors:** Julie A. Neumann, Grant E. Garrigues

**Affiliations:** Department of Orthopaedic Surgery, Duke University Medical Center, Durham, NC 27710, USA

## Abstract

Synovial chondromatosis is an uncommon condition, and involvement of the shoulder is even more rare. We report on a 39-year-old female who presented with symptoms, radiographic features, and intraoperative findings consistent with multiple subacromial loose bodies resulting in a partial-thickness, bursal-sided rotator cuff tear of the supraspinatus muscle. She was treated with an arthroscopic removal of loose bodies, complete excision of the subacromial/subdeltoid bursa, acromioplasty, and rotator cuff repair. To our knowledge, this is the first report of arthroscopic treatment for a bursal-sided, partial-thickness rotator cuff tear treated with greater than two-year clinical and radiographic follow-up. We utilized shoulder scores, preoperative and postoperative range of motion, and imaging to assess the results of treatment and surveillance for recurrence in our patient after two-year follow-up.

## 1. Introduction

Synovial chondromatosis (SC) is a rare monoarticular arthropathy with pathology characterized by foci of synovial chondroid metaplasia [1–3]. The foci of chondral tissue become pedunculated and eventually detach to form loose bodies within any synovial-lined potential space such as a diarthrodial joint, bursa, or tendon sheath [4, 5]. Once loose from the synovium, the cartilaginous bodies can continue to grow, nourished by synovial fluid. The loose bodies may undergo ossification, can create symptoms via their mass effect, and, in some cases, can cause periarticular or intra-articular erosive damage [5]. SC is not a true neoplastic condition [2] and its etiology is unknown [3, 4]. There have been numerous case reports to date involving knee, hip, elbow, wrist, ankle, and, least commonly, shoulder [3]. Traditional treatment consisted of an open arthrotomy with a subtotal synovectomy and removal of loose bodies [5]. More recently, arthroscopic techniques have been described. To our knowledge, only a handful of cases of bursal-sided rotator cuff tears associated with SC have been reported in the literature. These have generally had short follow-up and no radiographic assessment for disease recurrence. The current case report and literature review involve arthroscopic treatment of bursal-sided, partial-thickness supraspinatus tear with two-year follow-up.

## 2. Case Report

A 39-year-old, right-hand-dominant female presented with a five-year history of left shoulder pain, acutely worsening in the four to five months prior to presentation. She denied loss of motion, weakness, or any preceding left shoulder trauma. She reported pain at rest and with activity, especially overhead motion, and occasionally awaking her from sleep. She denied constitutional symptoms. Her past medical history was remarkable only for active cigarette use with a 25-pack-year smoking history.

Physical examination revealed no erythema, warmth, drainage, or muscle atrophy about the left shoulder. Active motion of the involved (left) shoulder for forward flexion, external rotation, and internal rotation measured 150 degrees, 70 degrees, and T10 (10th thoracic vertebra), respectively. She reported pain at the ends of all planes of range of motion. On the right side her active motion was 180 degrees, 70 degrees, and T12. On the right she had full painless active range of motion. Her left side had no detectable rotator cuff weakness. Neer and Hawkins-Kennedy impingement tests elicited pain. She had no crepitus with range of motion.

**Figure 1:**
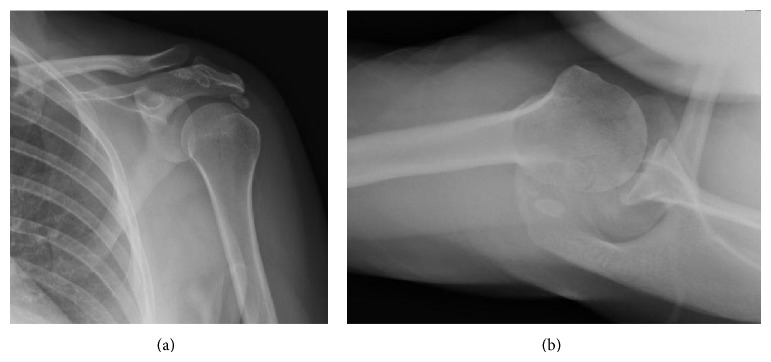
Preoperative (a) anteroposterior and (b) axillary lateral radiographs of the left shoulder showing periarticular calcified nodules and erosion of the AC joint.

**Figure 2:**
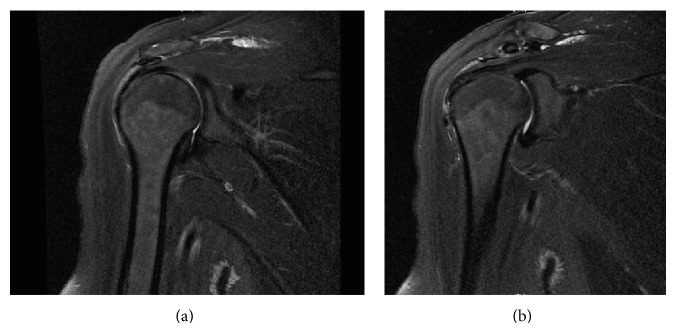
Preoperative T2-weighted, coronal oblique MRI showing (a) a small, nearly full-thickness tear of anterior supraspinatus tendon as well as (b) multiple extratendinous ossific densities consistent with synovial chondromatosis.

Plain radiographs (Figure 1) of the left shoulder were interpreted by a musculoskeletal trained radiologist as calcific densities within the rotator cuff versus extratendinous loose bodies, as well as erosion of the acromioclavicular (AC) joint. Magnetic resonance imaging (MRI) was ordered to accurately assess the exact location of the calcific nodules. MRI (Figure 2) demonstrated multiple extratendinous ossific densities in the subacromial/subdeltoid bursa, consistent with synovial chondromatosis. MRI also demonstrated fraying of the anterior supraspinatus tendon with minimal tendon retraction suggesting a small, full-thickness tear.

**Figure 3:**
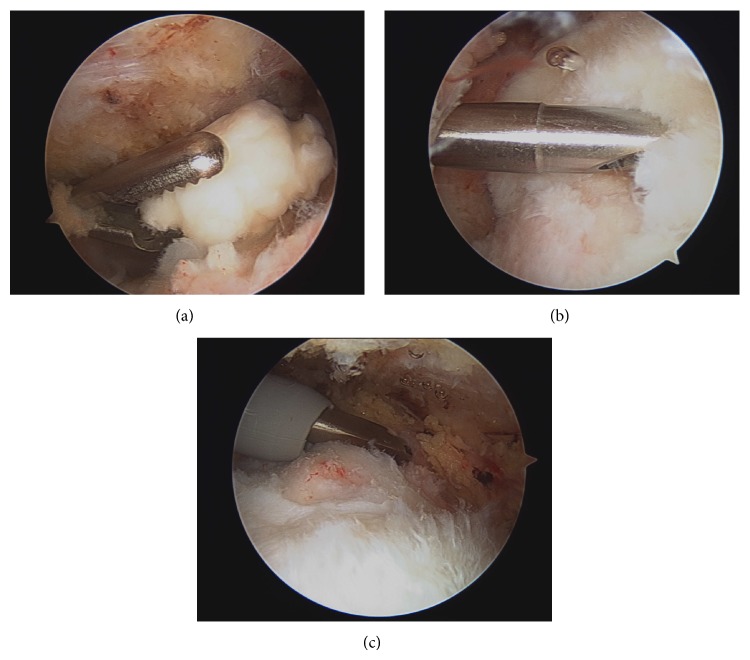
Arthroscopic view into the subacromial space showing (a) large subacromial loose body and ((b), (c)) 7-8 mm deep, partial-thickness, bursal-sided supraspinatus tear.

It was felt that conservative management would not be beneficial. Therefore, the patient underwent a diagnostic shoulder arthroscopy in the sitting position. A standard posterior portal was established and findings within the glenohumeral joint included normal appearing biceps tendon and pulley, intact rotator cuff from the articular side, no synovitis, intact labrum, and normal-appearing chondral surfaces of both the glenoid and humeral head. There were no loose bodies. The subacromial and subdeltoid space was then examined with bursoscopy, initially via the same posterior skin incision. Findings included extremely adherent and inflamed bursal tissue, partial-thickness, bursal-sided supraspinatus rotator cuff tearing without retraction, and more than 10 osseous bodies. Some of these cartilaginous bodies were adherent to the synovium and others were loose in bursae (Figure 3). One of the chondral bodies was adherent to the synovium and placed directly between the acromion and the greater tuberosity at the location of the rotator cuff tear. Several of the cartilaginous bodies were over 1.5 cm in diameter.

The patient underwent arthroscopic removal of all cartilaginous loose bodies, complete resection of the subacromial/subdeltoid synovial bursa, acromioplasty, and double row supraspinatus repair. Viewing from posterior, lateral, and anterior vantages facilitated removal of all loose bodies and bursal resection. After debridement of the footprint, an 80% thickness bursal-sided tear was observed. The remaining articular-sided fibers were left intact and a 5.5 mm double loaded BioComposite Corkscrew Anchor (Arthrex; Naples, Florida) was placed lateral to the intact tendon. Forty-five degree left and right curved lassos were used to pass both sutures through the tendon in a horizontal mattress fashion. The sutures were tied with alternating half-hitches on alternating posts and the tails were bought over to a 4.75 mm BioComposite SwiveLock anchor (Arthrex; Naples, Florida) laterally for a transosseous equivalent repair technique.

**Figure 4:**
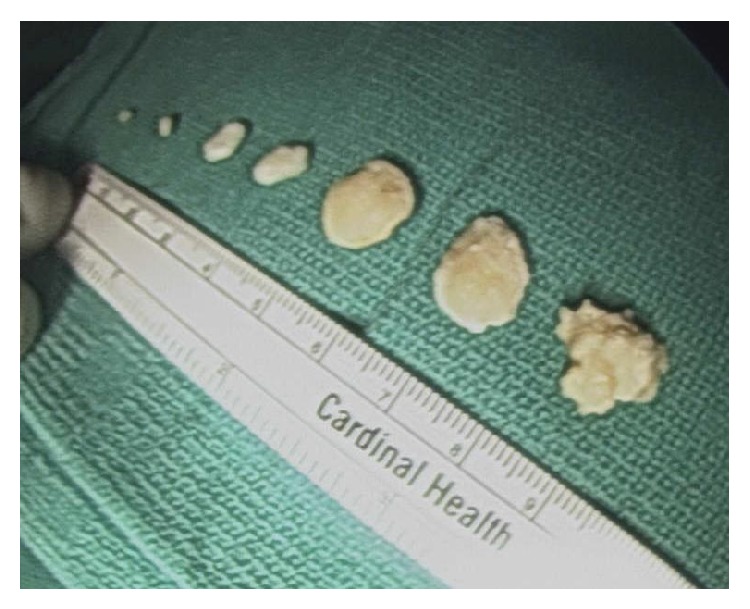
Multiple cartilaginous bodies of various sizes after arthroscopic removal.

Mini C-arm fluoroscopy was used to assess that all osseous loose bodies were removed. No additional calcified bodies were identified. Pathology was consistent with synovial chondromatosis with multiple cartilaginous loose bodies throughout the bursa (Figure 4).

**Figure 5:**
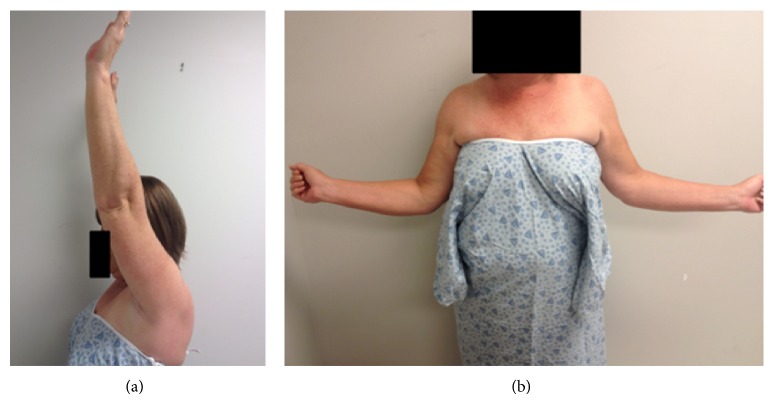
Her range of motion at 24 months postoperatively was 165 degrees in forward flexion (a), 75 degrees in external rotation (b), and T9 in internal rotation.

Postoperatively, the patient was placed in a sling-immobilizer and underwent routine rotator cuff repair physical therapy. Two years after surgery, she denied symptoms of residual loose bodies including impingement or crepitus. Repeat radiographs showed no recurrence of detectable osteochondromatosis. She continued to smoke one pack per day although she was counseled against this. The American Shoulder and Elbow Surgeons (ASES) Evaluation Form score was 95/100, SF-12 physical component summary was 51.3, mental component summary was 54.2, the Simple Shoulder Test (SST) was 9/12, and the Single Assessment Numeric Evaluation (SANE) score was 90 [6, 7]. Her active ranges of motion at 24 months after surgery for forward flexion, external rotation, and internal rotation were 165 degrees, 75 degrees, and T9, respectively (Figure 5). She had no pain at rest or with range of motion.

## 3. Discussion

Multiple case reports describe synovial chondromatosis in the knee, hip, elbow, wrist, ankle, and shoulder [8] with the shoulder being the least common of these joints to be affected [5]. Bloom and Pattinson demonstrated that the shoulder was involved in only 10 of 191 patients with SC [4] and Milgram and Hadesman noted shoulder involvement in six out of 30 patients [9]. Maurice et al. reviewed 53 patients and found no shoulder involvement [10]. Of the shoulder cases, involvement of the bursae is considerably more rare [3, 9]. In 1988 Milgram and Hadesman were the first to describe bursal-sided osteochondromatosis of the shoulder [9]. To date, only a handful of cases have been described in which synovial chondromatosis results in partial [3] or complete bursal-sided rotator cuff tears (Table 1) [11], none of which have complete follow-up data. We believe that the etiology of the bursal-sided, partial-thickness rotator cuff tear described in this case was SC; however, it may have been a concomitant rotator cuff tear. There have been three reports of full-thickness rotator cuff tearing felt to be secondary to subacromial synovial chondromatosis [2, 8, 11]. Horii et al. described bursal-sided, partial thickness tears secondary to subacromial SC [8, 12]. Ogawa et al. reported a patient with bilateral, bursal-sided, partial thickness rotator cuff tears thought to be due to synovial osteochondromatosis in bilateral subacromial bursae [3]. All six instances of subacromial synovial chondromatosis and concomitant rotator cuff tears were treated with loose body removal, six with acromioplasty, five with rotator cuff repair (two open, one arthroscopic, and two unspecified), and one with distal clavicle excision. All had outcomes typical for rotator cuff repair with improvements in subjective strength, active range of motion, and pain. See Table 1 for a description and outcome of each of these six cases.

In our patient, radiographs and MRI were used to detect the loose bodies. Calcifications can develop months to years after clinical symptoms; thus caution should be utilized when using only radiographs for diagnosis [13, 14]. Cross-sectional imaging, including MRI and computerized tomography, can be used to confirm the diagnosis in early phases of disease [1, 8, 13, 14]. Loose bodies usually display a low signal on T1-weighted images and a high signal on T2-weighted images, typical of the high water content of cartilage [1].

Exact timing of surgical intervention has not been defined in the literature [15], but it is hypothesized that loose bodies within the subacromial space may lead to supraspinatus outlet impingement, acromial spurring, and bursal-sided rotator cuff tears [2]. For this patient, surgical treatment was pursued to provide pain relief as well as to prevent further damage to the rotator cuff from subacromial impingement or the AC joint from periarticular erosions. Recurrence rates following open and arthroscopic treatment of shoulder SC are comparable (0–31%) [1, 8]. Generally, arthroscopic treatment results in low morbidity, earlier return to function, shorter rehabilitation course, decreased postoperative pain, and earlier active range of motion [8, 15, 16] when compared to open treatment. The entire subacromial bursa, synovium, and all of the loose bodies were arthroscopically removed from this patient. To date, it is unclear whether a synovectomy should be performed in conjunction with loose body removal. Jeffreys [16] and Paul and Leach [17] each concluded that additional synovectomy offers advantage to simply removing loose bodies [15] and this was supported by Dorfmann et al.'s research in knees [18]. Ogilvie-Harris and Saleh favor synovectomy because “persistent metaplastic activity of the synovium leads to recurrence” [19]. Histopathologic analysis of loose bodies is recommended to monitor for rare malignant transformation [5].

To our knowledge, only one other report describes greater than two-year follow-up of a patient with bursal-sided, partial-thickness rotator cuff tears secondary to SC treated arthroscopically [3]. Our results are unique in that we utilized validated shoulder outcome scores, preoperative and postoperative range of motion, and imaging to assess the results of treatment and surveillance for recurrence after two-year follow-up.

In conclusion, we have reported on a patient with synovial chondromatosis of the subacromial bursae that resulted in impingement as well as a bursal-sided, partial-thickness rotator cuff tear. This appears to be an example of true “impingement syndrome” creating a rotator cuff tear due to mechanical wear on the underlying rotator cuff. Plain films suggested the diagnosis and MRI revealed the additional finding of bursal-sided rotator cuff tearing. The patient was treated successfully using arthroscopy for complete subacromial bursectomy, removal of loose bodies, and rotator cuff repair. The patient had a satisfactory clinical outcome and no recurrence at two-year follow-up.

## Figures and Tables

**Table 1:** Outcomes of bursal-sided rotator cuff tears secondary to SC.

Authors, year	Site of RCT	Thickness/size of RCT	Open versus arthroscopic	Procedure	Follow-up (months)	Outcome
Milgram and Hadesman, 1988 [9]	Unspecified	Unspecified	Open (deltopectoral)	LBR, RCE, acromioplasty, DCE, and SAD	6	Recovered shoulder function over a two-month period

Ko et al., 1995 [12]	Supraspinatus, infraspinatus	4 full-thickness tears and multiple partial-thickness tears which were slit-like and perpendicular to axis of tendons	Open (Saber)	LBR, RCE with direct sutures, acromioplasty, and partial synovectomy	24	No symptoms, normal ROM, and radiographs without recurrence

Ogawa et al., 1999 [3]	Supraspinatus, bilateral	Bursal-sided, partial-thickness tear, 5 mm in depth	Arthroscopic	LBR, RCR, acromioplasty, and bursectomy	48	Only mild pain when engaging in sports and no radiographs reported

Horii et al., 2001 [11], case 1	Unspecified	Partial-thickness, longitudinal tear	Unspecified	LBR, RCR with side-to-side suture, acromioplasty, and bursectomy	2	Returned to work without pain

Horii et al., 2001 [11], case 2	Unspecified	Partial-thickness, longitudinal tear 1 cm in length	Unspecified	LBR, RCR with side-to-side suture, acromioplasty, and bursectomy	12	Little pain and slight limitation in passive abduction at 130°

Huang et al., 2004 [2]	Supraspinatus	Full-thickness tear about 1 cm at the critical zone	Unspecified	LBR, RCR, and acromioplasty	12	Painless; AROM FF 170°, ER 30°, and IR T12. PROM FF 175°, ER 40°, and IR T10

RCT: rotator cuff tear.

LBR: loose body removal.

DCE: distal clavicle excision.

ROM: range of motion.

FF: forward flexion.

ER: external rotation.

IR: internal rotation.
